# Combined Metabolomic Analysis of Plasma and Tissue Reveals a Prognostic Risk Score System and Metabolic Dysregulation in Esophageal Squamous Cell Carcinoma

**DOI:** 10.3389/fonc.2020.01545

**Published:** 2020-08-26

**Authors:** Zhongjian Chen, Yalan Dai, Xiancong Huang, Keke Chen, Yun Gao, Na Li, Ding Wang, Aiping Chen, Qingxia Yang, Yanjun Hong, Su Zeng, Weimin Mao

**Affiliations:** ^1^College of Pharmaceutical Sciences, Zhejiang University, Hangzhou, China; ^2^Institute of Cancer and Basic Medicine (ICBM), Chinese Academy of Sciences, Hangzhou, China; ^3^Cancer Hospital of the University of Chinese Academy of Sciences, Hangzhou, China; ^4^Zhejiang Cancer Hospital, Hangzhou, China; ^5^School of Medicine, Imperial College London, London, United Kingdom; ^6^Department of Chemistry, Hong Kong Baptist University, Hong Kong, China

**Keywords:** esophageal squamous cell carcinoma (ESCC), metabolomics, risk score, prognosis, diagnosis, indoleamine 2, 3-dioxygenase 1 (IDO1), artificial intelligence

## Abstract

**Background:** Esophageal squamous cell carcinoma (ESCC) is a gastrointestinal malignancy with a poor prognosis. Although studies have shown metabolic reprogramming to be linked to ESCC development, no prognostic metabolic biomarkers or potential therapeutic metabolic targets have been identified.

**Method:** The present study investigated some circulating metabolites associated with overall survival in 276 curatively resected ESCC patients using liquid chromatography/mass spectrometry metabolomics and Kaplan-Meier analysis. Tissue metabolomic analysis of 23-paired ESCC tissue samples was performed to discover metabolic dysregulation in ESCC cancerous tissue. A method consisting of support vector machine recursive feature elimination and LIMMA differential expression analysis was utilized to select promising feature genes within transcriptomic data from 179-paired ESCC tissue samples. Joint pathway analysis with genes and metabolites identified relevant metabolic pathways and targets for ESCC.

**Results:** Four metabolites, kynurenine, 1-myristoyl-glycero-3-phosphocholine (LPC(14:0)sn-1), 2-piperidinone, and hippuric acid, were identified as prognostic factors in the preoperative plasma from ESCC patients. A risk score consisting of kynurenine and LPC(14:0)sn-1 significantly improved the prognostic performance of the tumor-node-metastasis staging system and was able to stratify risk for ESCC. Combined tissue metabolomic analysis and support vector machine recursive feature elimination gene selection revealed dysregulated kynurenine pathway as an important metabolic feature of ESCC, including accumulation of tryptophan, formylkynurenine, and kynurenine, as well as up-regulated indoleamine 2,3-dioxygenase 1 in ESCC cancerous tissue.

**Conclusions:** This work identified for the first time four potential prognostic circulating metabolites. In addition, kynurenine pathway metabolism was shown to be up-regulated tryptophan-kynurenine metabolism in ESCC. Results not only provide a metabolite-based risk score system for prognosis, but also improve the understanding of the molecular basis of ESCC onset and progression, and as well as novel potential therapeutic targets for ESCC.

## Introduction

Esophageal cancer (EC), a common gastrointestinal malignancy, ranks as the sixth leading cause of cancer death worldwide (1–3). EC is the fifth most common cancer and the fourth leading cause of death in China (4). Esophageal squamous cell carcinoma (ESCC) is the predominant histological subtype of EC in China (5, 6). Surgical resection with lymphadenectomy is the main treatment for ESCC (7). However, despite advances in surgical management and multidisciplinary treatment of ESCC, prognosis remains poor (8). Currently, tumor-node-metastasis (TNM) staging system is used for ESCC prognosis, even though staging components such as lymph node metastasis, invasion depth, and differentiation are not obtained during surgery but commonly determined postoperatively. Therefore, there is an urgent need for non-invasive and convenient biomarkers that may assist the clinical decision-making and provide novel insights into tumorigenesis and biology of ESCC (9).

Metabolic reprogramming is an oncogene-driven mechanism that alters the metabolism of cancer cells. It supports tumor proliferation and anabolic growth and is considered as an essential hallmark of cancer (10). Metabolomics, in which small-molecule metabolites are identified and quantified, is the closest “omics” to phenotype (11). Compared to other wide-ranging forms of analysis, metabolomics is more sensitive to alterations of biochemical homeostasis, providing comprehensive and direct information regarding cell status and treatment response. Metabolomic analysis requires a little sample material and preparation time (11). A growing body of literature demonstrates that monitoring cellular metabolites may not only provide promising biomarkers, but may also help to identify involved biological processes (11–13). For example, diagnostic and prognostic cancer biomarkers have been recently investigated in lung, colorectal, and breast cancers by different metabolomic approaches (14–16). Metabolomics, the study of altered metabolites accompanying cancer-associated metabolic reprogramming, is an emerging field that can contribute to the identification of novel cancer biomarkers and the discovery of potential drugs for prevention and therapy (17).

Previous metabolomic studies have demonstrated various metabolic alterations in patients with ESCC including changes in amino acids, glucose, lipids, organic acids, nucleotides, and fatty acids (18–21). Though many promising serum/plasma metabolites have been found to be diagnostic biomarkers for ESCC (18, 22, 23), no metabolite with prognostic value has been identified, nor has a potential metabolic therapeutic target been recognized. Gu et al. found serum D-mannose to be a novel prognostic biomarker for patients with esophageal adenocarcinoma (the main histological subtype in the USA). Those results encouraged us to investigate potential ESCC prognostic circulating metabolites by a combination metabolomics and survival analysis (24).

**Figure 1:**
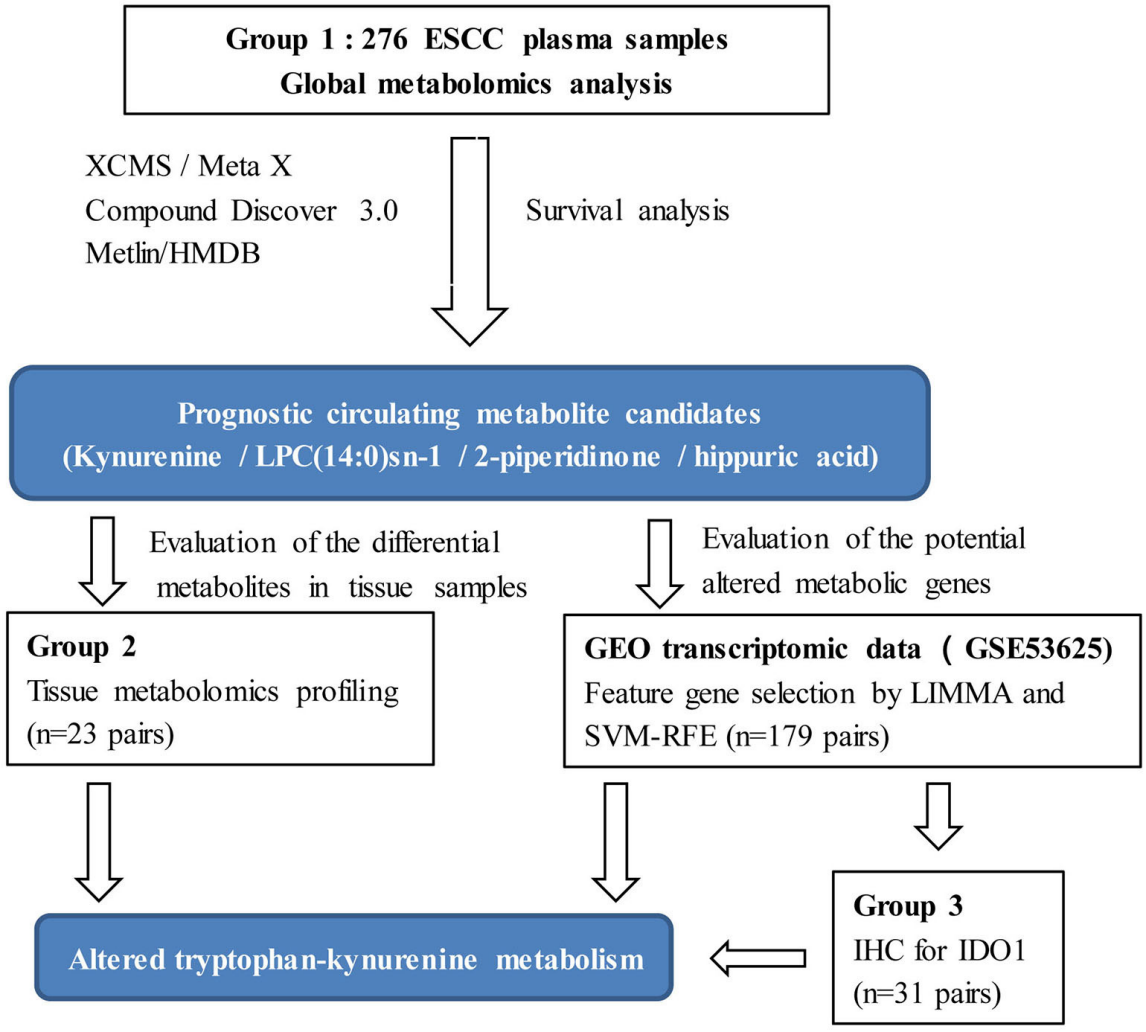
Flowchart of this study.

Despite the advantages of metabolomics, limitations for clinical application need to be considered. Due to the dynamic and sensitive nature of the metabolome, clinical metabolomic studies in particular, must be designed based on a relatively large sample size to reduce unwanted excessive variability, and results validated by multiple models or sample types (25). Thus, the aim of the present study was to discover metabolic biomarkers and potential metabolic therapeutic targets for ESCC with the following design improvements. (1) Plasma metabolic profiling was conducted on a relatively large sample size (*n* = 276). (2) Prognostic metabolites were discovered by survival analysis. (3) Integrative bioinformatics and metabolomics were used to discover metabolic features and potential therapeutic target enzymes for ESCC (Figure 1). Our results will assist clinicians in management of ESCC patients, as well as contribute to an understanding of the mechanisms underlying ESCC tumorigenesis, and possibly offer novel therapeutic targets.

## Materials and Methods

### Chemicals and Reagents

Acetonitrile (high-performance liquid chromatography (HPLC) grade) and Methanol (HPLC) were purchased from Tedia (Ohio, USA). Formic acid (HPLC) was purchased from Roe Scientific Inc. (Delaware, USA). Distilled water was from Wahaha Group Co., Ltd. (Hangzhou, China). L-kynurenine (purity > 98%), L-tryptophan (purity > 98%), and hippuric acid (purity > 98%) were purchased from Sigma-Aldrich (Missouri, USA). L-phenylalanine (purity > 98%), 2-piperidinone (purity > 98%), and LPC(14:0)sn-1 (purity > 98%) were purchased from Aladdin Reagent Co. Ltd. (Shanghai, China). Rabbit anti-indoleamine 2, 3-dioxygenase 1 (IDO1) polyclonal antibody (13268-1-AP) was purchased from Proteintech Group, Inc. (Hubei, China).

### Study Patients and Samples

Group 1: the preoperative fasting plasma samples, using the di-potassium salt of ethylenediaminetetraacetic acid as anticoagulant, were collected from 276 patients recruited after histopathologic confirmation of ESCC and radical resection at Zhejiang Cancer Hospital (Hangzhou, China), from May 2010 to December 2012. Clinicopathological features and preoperative biochemical parameters were obtained. Participants were followed until December 2017, evaluating overall survival (OS) from surgery to the date of death or of the last follow-up visit. Group 2: a total of 23-pairs of matched cancerous and normal tissue samples were used for tissue metabolomic study. Normal tissues were collected from the distal edge of the resected tissues, at more than 2 cm from the solid tumor border. Group 3: a total of 31-pairs of matched cancerous and normal tissue samples were assessed by immunohistochemistry (IHC). All samples collected in this study were stored at −80°C until analysis. Demographic and clinicopathologic characteristics of the patients are reported in Table 1.

**Table 1:** Demographic and clinicopathologic characteristics of study patients.

**Parameters^**a**^**	**Group 1^**b**^**	**Group 2^**b**^**	**Group 3^**b**^**
	***n* = 276**	***n* = 23 (paired)**	***n* = 31 (paired)**
**Sex**			
Male	232	19	3
Female	44	4	28
**Age**			
Mean age, yr	60.8 ± 7.1	59.7 ± 7.3	62.0 ± 6.7
**BMI**			
Median(range)	22 (16-30)	22 (17-27)	22 (16-28)
**Smoking habit**			
No	79	8	10
Yes	197	15	21
**Alcohol consumption**			
No	88	10	10
Yes	188	13	21
**Tumor thrombus**			
No	191	16	20
Yes	85	7	11
**Neural invasion**			
No	153	16	21
Yes	123	7	10
**Tumor grade**			
Well	15	1	3
Moderately	186	14	16
Poorly	75	8	12
**N stage**			
0	101	11	12
1	99	8	13
2	49	3	5
3	27	1	1
**T stage**			
1	12	3	4
2	49	3	3
3	213	17	24
4	2	-	-
**TNM stage**			
1	23	4	4
2	85	7	9
3	144	11	17
4	24	1	1

a *N stage, T stage, and TNM stage were determined according to the American Joint Committee on Cancer, 8th edition*.

b *Group 1: Preoperative plasma samples from 276 patients with ESCC were used for plasma metabolomics. Group 2: 23-pairs of matched cancerous and normal tissue samples were used for tissue metabolomic study. Group 3: 31-pairs of matched cancerous and normal tissue samples were assessed by IHC analysis*.

The study protocol was performed in accordance with the declaration of Helsinki, approved by the Research Ethics Committee of Zhejiang Cancer Hospital, China, with written informed consent obtained from all individuals.

### Plasma-Based Metabolomic Analysis

#### Sample Preparation

Plasma samples were from Group1 (Table 1). Plasma samples (50 μL) were thawed on ice and immediately mixed with 200 μL of ice-cold acetonitrile. After mixing by vortex for 1 min, the mixture was centrifuged at 16,200 g for 15 min at 4°C. The supernatant (150 μL) was transferred into a fresh tube and lyophilized. The residues were resuspended by adding 80 μL of 25% acetonitrile in water and mixed by vortex for 1 min. After centrifugation at 16,200 g for 15 min at 4°C, 60 μL of the supernatant was transferred into the sample bottle. A supernatant aliquot of 10 μL was used for liquid chromatography-mass spectrometry (LC-MS) analysis.

Quality control (QC) samples were prepared by pooling the re-dissolved sample with an equal amount (15 μL) and periodically analyzed throughout the complete analytical run to monitor signal drift.

#### LC-MS Analysis

LC-MS analysis was conducted as previously described (26). The Ultimate 3000 UHPLC system (Dionex, Idstein, Germany), linked to a Q Exactive orbitrap mass Spectrometer (Thermo Fisher Scientific, Bremen, Germany), was used. Separation was performed on an ACQUITY UPLC HSS T3 column (2.1 mm × 100 mm × 1.8 μm, Waters, MA, USA) at 35°C, with a mobile phase consisting of 0.1% formic acid and acetonitrile. The gradient is reported in Table S1. Full mass scan mode was used for all the samples and data-dependent MS/MS acquisition mode was utilized for the identification of QC samples. Detailed parameters are listed in Table S2.

#### Metabolomic Analysis

Raw data were converted to mzXML format using MSconvert program (http://proteowizard.sourceforge.net/download.html). The R package *XCMS* (version 3.3.2) was utilized for data preprocessing, including retention time alignment, peak detection, and peak matching. R package *MetaX* (version 1.4.16) was used to remove peaks with more than 20% of zero values in all samples or the peaks with coefficient of variation values >30% in QC. Peaks were corrected with the QC-robust LOESS signal correction algorithm. All the detected ions in each sample were normalized to the sum of the peak area defined as 100,000 (27). Thermo Scientific Compound Discoverer 3.0 software (Thermo Fisher Scientific, USA) combined with the METLIN (http://metlin.scripps.edu) and the HMDB (http://www.hmdb.ca/) databases were used for metabolite annotation by comparison of MS fragmentation information. Standard substances were used to verify prognostic metabolites.

#### Survival Analysis With Regard to Circulating Metabolites

Once relative concentrations of circulating metabolites were obtained by metabolomic analysis, Kaplan-Meier curves were performed to identify associations between metabolite levels and OS, with median split and log-rank test. Cox proportional hazards regression test was also performed for each metabolite to calculate the hazard ratio (HR) value. Factors with *P*-values < 0.05 were considered to have prognostic significance. Multivariate Cox proportional hazards regression was analyzed to estimate independent and significantly prognostic circulating metabolites. With the independently prognostic metabolites, a risk score was derived by summation of each metabolite level multiplying their corresponding coefficient according to Li et al. (28).

### Tissue-Based Metabolomic Analysis

#### Sample Preparation and LC-MS Analysis

Tissue samples were from Group 2 (Table 1). Approximately 20 mg of tissue was transferred into a 1.5 mL tube with immediate addition of 400 μL of ice-cold methanol and two steel balls (diameter: 2 mm). Homogenization was performed with a Tissuelyser (2 min, 30 Hz). After centrifugation at 16,200 g for 15 min at 4°C, 200 μL of the supernatant was transferred into a fresh tube, to which was added with 200 μL of water followed by lyophilization. Reconstitution, analysis protocols as well as QC sample preparation were conducted by the same methods used for plasma-based metabolomics. The LC-MS analysis and metabolomic analysis protocols were the same as that for plasma-based metabolomics.

#### Metabolomic Analysis

Relative concentrations of ion features were obtained from metabolomic data with the same protocol as that for plasma-based metabolomics. Unsupervised principal component analysis (PCA) was conducted to assess the trends for all samples. Supervised partial least squares discriminant analysis (PLS-DA) was performed to identify the most discriminating ion features between ESCC cancerous tissues and non-cancerous counterparts based on variable importance in projection (VIP) values. Finally, ions with VIP > 1, Benjamini–Hochberg adjusted *p*-value (FDR) < 0.05, and |log2(Fold change)| > 0.585 were defined as differential ion features. Metabolite annotation was performed using the above method. Receiver operating characteristic (ROC) curve analysis was used to evaluate the diagnostic significance of metabolites, in order to distinguish ESCC cancerous and non-cancerous tissues.

### Bioinformatic Analysis

The ESCC microarray dataset (GSE53625) was generated using the Agilent-038314 CBC *Homo sapiens* lncRNA+mRNA microarray V2.0 (http://www.genomics.agilent.com/) deposited in the Gene Expression Omnibus (GEO) (https://www.ncbi.nlm.nih.gov/geo/) and processed as described previously (9). Briefly, probe re-annotation was performed based on the previous study (29). For genes with multiple probes, mean expression was calculated and used. LIMMA (R package version 3.38.3) was used to analyze differentially expressed genes (DEGs) and genes with |log_2_(Fold change)| > 1 and FDR < 0.05 were considered to be DEGs. The differential DEGs were further ranked by support vector machine recursive feature elimination (SVM-RFE) algorithm proposed according the Huang et al. (30). Briefly, the SVM-RFE computes the ranking weights for all DEGs and sorts the DEGs according to weight vectors as the classification basis. SVM-RFE conducted an iteration process of the backward removal of DEGs as follows: (1) use the current dataset to train the classifier; (2) calculate the ranking weights for all DEGs; (3) eliminate the DEG with the smallest weight. The above iteration process repeats until there is only one DEG remained in the dataset, and the implementation result provides a list of DEGs in the descending order of weight. Top 500 genes were selected as features for further analysis. Additionally, the metabolic feature genes were obtained through searching on KEGG (31). Then, pathway analysis was conducted by searching genes and metabolites together by KEGG Mapper Search (https://www.genome.jp/kegg/mapper.html). The metabolism pathway/module for both hits from gene and metabolite was considered to be of potential importance.

### IHC Analysis

Tissue sections (4 μm thick) were dewaxed and rehydrated through graded alcohols. IHC staining with IDO1 antibodies was performed according to the manufacturer's instructions. The results were analyzed using a semiquantitative method (32), with the immunohistochemical score calculated by multiplying the percentage of positive cells by staining intensity.

### Statistical Analysis

Statistical analysis was performed using SPSS 16.0 software (USA) and R software (http://www.r-project.org/). Normality of the variables was tested by Shapiro-Wilk normality test. Unpaired Wilcoxon rank-sum test, and Kruskal-Wallis test were used for comparison of two or more than two groups of data. The correlation between circulating metabolites and other variables was calculated by Kendall rank or Spearman's rank correlation. ROC analysis and ROC comparisons were performed by R package *pROC* (version 1.15.3). A two-tailed *p* value < 0.05 was considered statistically significant.

## Results

### Identification of Circulating Prognostic Metabolites for ESCC

**Figure 2:**
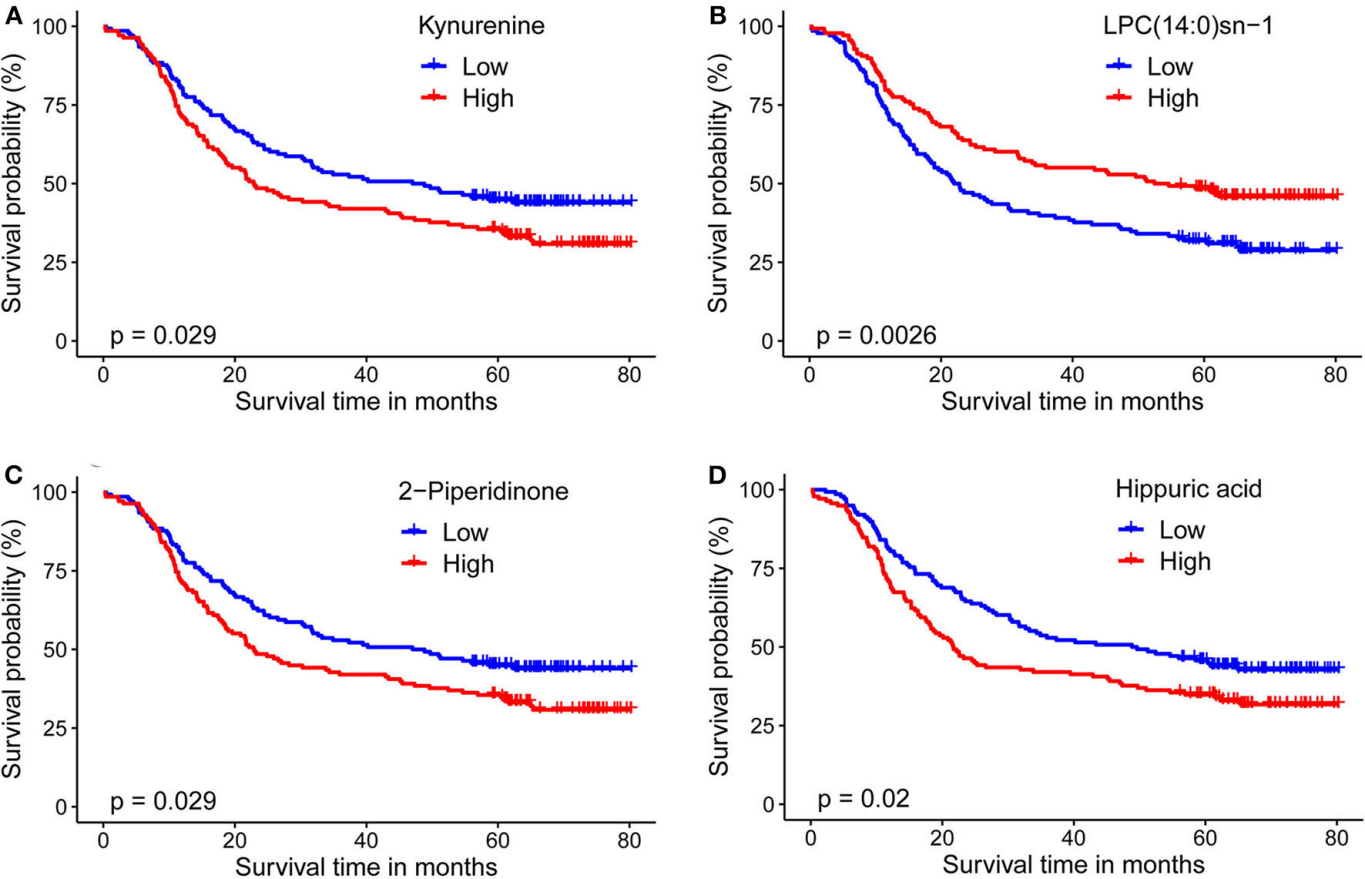
Kaplan-Meier survival curves for ESCC patients stratified by the four circulating metabolites by a median-split. **(A)** Kynurenine; **(B)** LPC(14:0)sn-1; **(C)** 2-Piperidinone; **(D)** Hippuric acid. Log-rank test was used, *p* < 0.05 was considered significant.

For plasma-based metabolomic profiling study (Group 1), a total of 4,121 metabolic features in electrospray ionization positive mode and 3,046 in electrospray ionization negative mode were extracted from the metabolomic data. A total of 129 ion features were annotated with metabolites (Tables S3, S4). Survival analysis aided identification of four metabolites with *p* values < 0.05: kynurenine, LPC(14:0)sn-1, 2-piperidinone, and hippuric acid. Higher plasma levels of kynurenine, 2-piperidinone, and hippuric acid correlated with shorter survival, while higher levels of LPC(14:0)sn-1 correlated with longer survival (Figures 2A–D, Table 2). Moreover, Multivariate Cox regression analysis of the four metabolites indicated that kynurenine and LPC(14:0)sn-1 to be independent factors suitable for metabolite-based risk score calculation (Table 2).

**Table 2:** Prognostic circulating metabolites in ESCC plasma.

**Metabolites**	**Features in LC/MS**			**KM^**d**^**	**Cox regression**^****e****^	
	**m/z^**a**^**	**RT (min)^**b**^**	**Ion^**c**^**	***p* value**	***p* value**	**HR (95%CI)**
2-Piperidinone	100.07629	3.9	Positive	0.035	0.2	1.22 (0.90-1.67)
Kynurenine	209.09222	3.9	Positive	0.029	0.035	1.40 (1.03-1.91)
LPC(14:0)sn-1	468.30879	8.9	Positive	0.0026	0.0019	0.61 (0.45-0.84)
Hippuric acid	178.05156	5.2	Negative	0.02	0.11	1.29 (0.95-1.76)

a *m/z, mass/charge number*;

b *RT, retention time*;

c *mass spectrometer scan types*;

d *KM, Kaplan-Meier*;

e *Cox regression analysis of 2-piperidinone, kynurenine, LPC(14:0)sn-1 and hippuric acid, and HR, hazard ratio*.

These four prognostic metabolites were compared with chromatograms and spectra of reference substances. A representative identification of kynurenine is shown in Figure S1, while the identification of the other three metabolites is illustrated in Figure S2.

In order to clarify the potential influence of demographic factors on these prognostic circulating metabolites, multivariate Cox regression was performed for each of the four metabolites with age, sex, smoking habit, and alcohol consumption. Result demonstrated that kynurenine (HR: 1.37, *p* = 0.040), LPC(14:0)sn-1 (HR: 0.618, *p* = 0.00229), hippuric acid (HR: 1.423, *p* = 0.021) were independent prognostic factors, while 2-piperidinone (HR: 1.35, *p* = 0.054) was not an independent factor.

### Potential Relationships Among Prognostic Metabolites, Clinicopathologic Features, and Biochemical Parameters

Analysis of the four circulating metabolites indicated that kynurenine was significantly positively correlated with the other three metabolites, while 2-piperidinone positively correlated with hippuric acid (Table S5). The four metabolites were assessed for correlations with clinicopathologic features including sex, age, body mass index (BMI), smoking habit, alcohol consumption, tumor grade, tumor thrombus, neural invasion, T stage, N stage, and TNM stage. The following biochemical parameters were also included in the correlation analysis: glycyl proline dipeptidyl aminopeptidase (GPDA), alanine aminotransferase (ALT), gamma-glutamyltransferase (GGT), prealbumin (PA), albumin (ALB), triglyceride (TG), total cholesterol (TC), low density lipoprotein cholesterol, high density lipoprotein cholesterol. The results of this analysis showed that: (1) Kynurenine levels were positively correlated with N stage and GGT levels, while a negative correlation was found with tumor grade and ALB levels. (2) LPC(14:0)sn-1 levels were positively correlated with GPDA, ALT, PA, and TG levels, and negatively correlated with age. (3) 2-piperidinone levels were positively correlated with GGT levels, BMI, and alcohol consumption, while it was negatively correlated with TC levels. (4) Hippuric acid levels were positively correlated with GPDA levels and negatively correlated with GGT, ALT, and PA levels (Table S6).

### Metabolite-Based Risk Score Improves Prognostic Performance

Cox proportional hazards regression analysis showed kynurenine and LPC(14:0)sn-1 regression coefficients of 0.41 and −0.52, respectively. A risk score was attributed to each patient by adding the plasma level of each metabolite multiplied by the corresponding regression coefficient: risk score = (0.409 × level of kynurenine −0.522 × level of LPC (14:0)sn-1) (28). The risk score of all cases was calculated according to this formula and the patients were stratified into low-risk and high-risk groups, by applying the median-split method. Risk score efficiently stratified ESCC risk (Figure S3) independent of TNM or N stage (Table S7).

**Figure 3:**
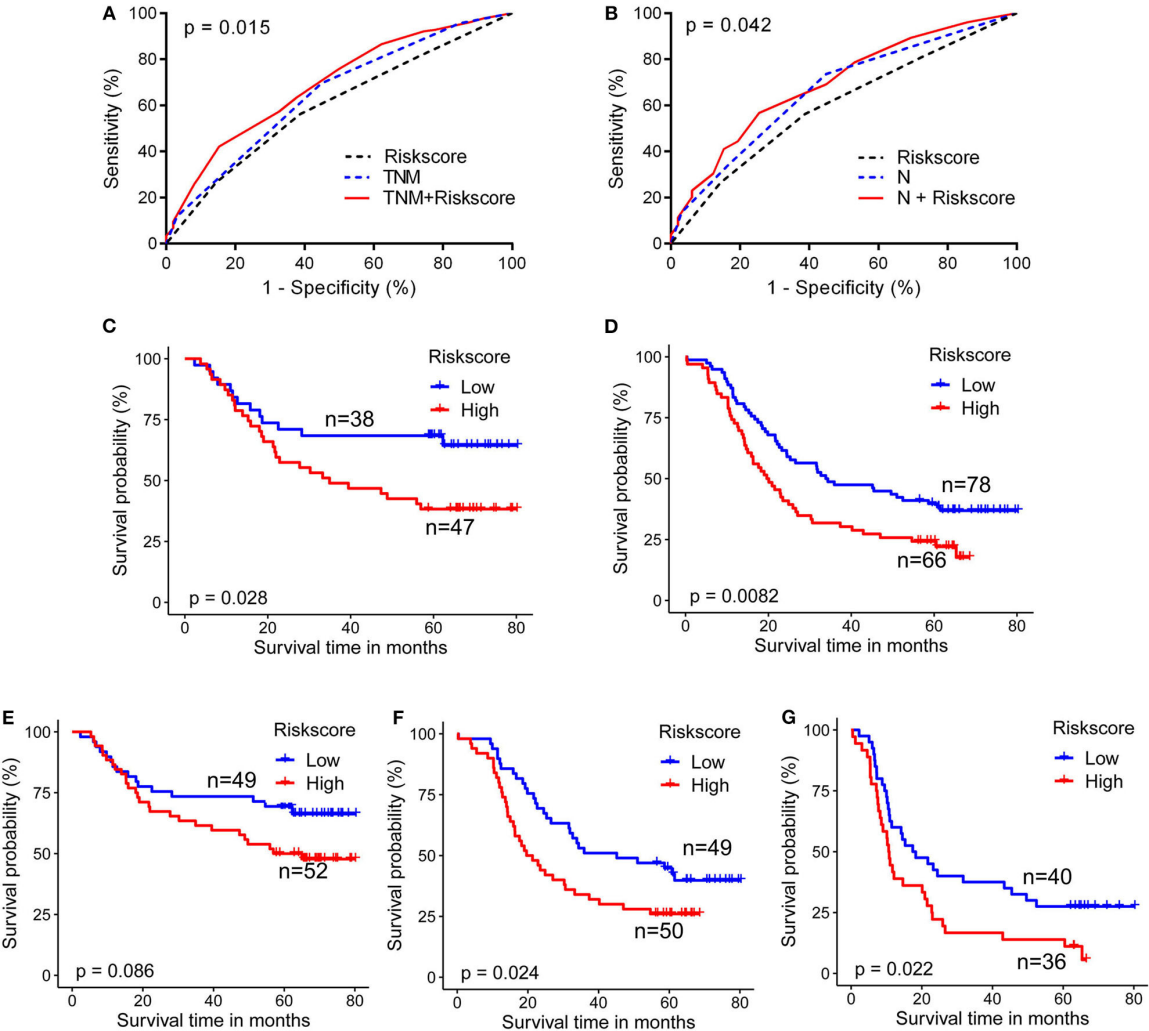
Metabolite-based risk score improves prognostic performance for ESCC patients. **(A)** Combination of risk score with TNM stage **(B)**, or with N stage (G) significantly improved the predictive accuracy of 5-year survival rate. ESCC cases classified according to TNM II **(C)**, TNM III **(D)**, N1 **(F)**, and N2 **(G)** stages were significantly stratified by risk score. Cases from N0 **(E)** showed a trend for stratification by risk score. DeLong test for AUC in ROC curves and log-rank test for Kaplan-Meier survival curves were assessed, *p* < 0.05 was considered significant.

Area under the curve (AUC) of ROC curves for 5-year survival status prediction was calculated and compared using the method established by DeLong et al. (33). When combined with the risk score staging classification, the prediction accuracy of the conventional TNM stage and N stage was significantly improved from 0.650 (95% confidence interval (CI): 0.583-0.718) to 0.692 (95% CI: 0.628-0.756; *p* = 0.015), and from 0.665 (95% CI: 0.599-0.731) to 0.694 (95%CI: 0.630-0.750; *p* = 0.042), respectively (Figures 3A,B). Moreover, log-rank analysis of Kaplan-Meier curves related to the metabolite-based risk score groups demonstrated that the calculated risk score was able to significantly improve the prediction of clinical outcome in patients with ESCC, classified according to the stages TNM II (*p* = 0.028), TNM III (*p* = 0.008), N1 (*p* = 0.024), and N2 (*p* = 0.022; Figures 3C,D,F,G), while for N0 classification a statistically non-significant (*p* = 0.086) trend to stratification based on the risk score was observed (Figure 3E).

### Tissue-Based Metabolomics Reveals Altered Kynurenine Pathway in ESCC

**Figure 4:**
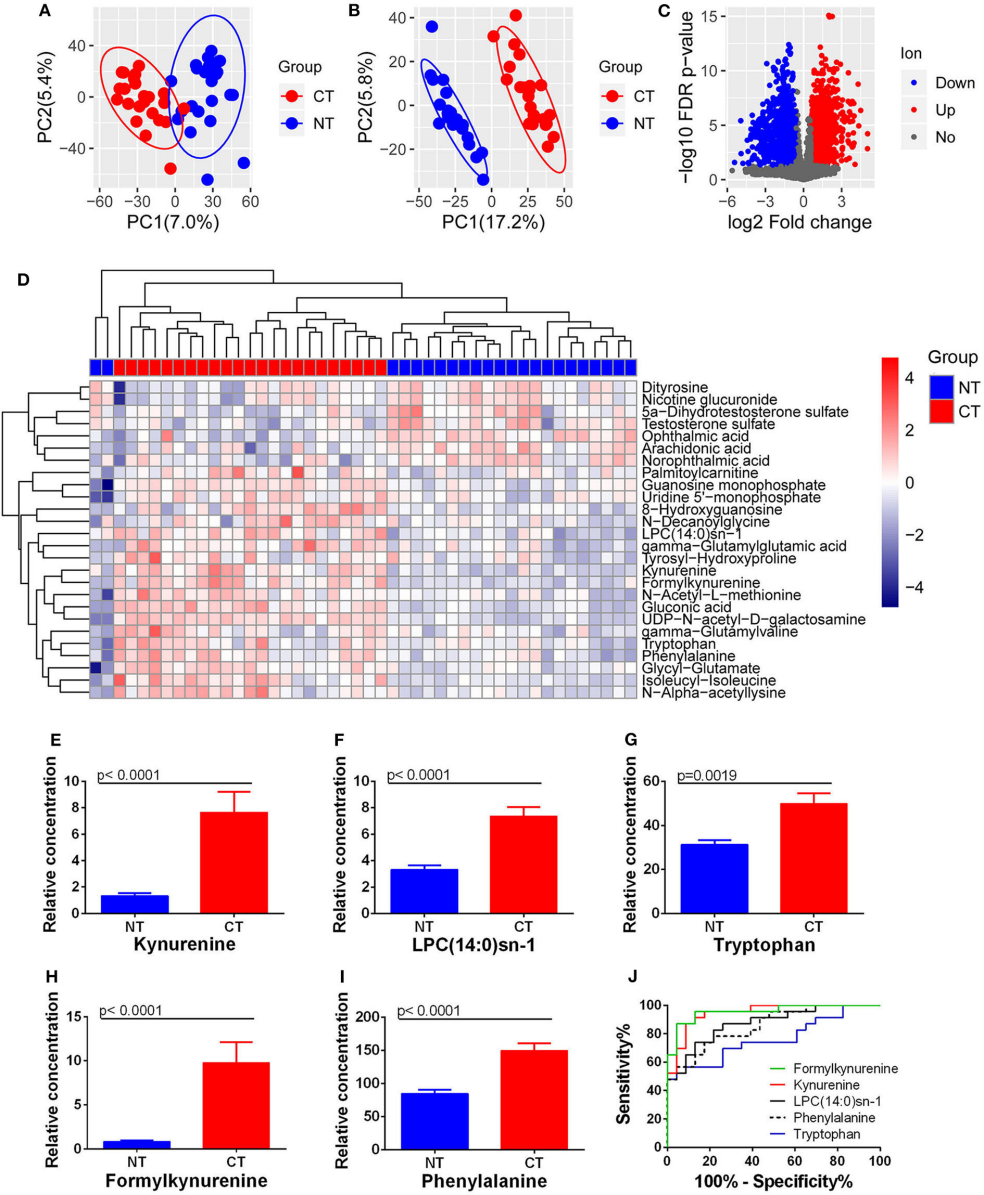
Changes in metabolites between ESCC cancerous (CT) and normal tissues (NT). **(A)** PCA and **(B)** PLS-DA analysis with all ion features. **(C)** Differential ion features were defined as |log2(Fold change)| > 0.585 and FDR < 0.05. **(D)** Heatmap analysis with 26 differential metabolites. Accumulation of kynurenine **(E)**, LPC(14:0)sn-1 **(F)**, tryptophan **(G)**, formylkynurenine **(H)**, and phenylalanine **(I)** was observed in ESCC cancerous tissues compared to normal equivalents. ROC curves of these metabolites showed the potential diagnostic value for ESCC **(J)**.

Both PCA and PLS-DA analysis with the extracted 4,856 ion features showed a significant metabolic shift between ESCC cancerous and normal tissues (Figures 4A,B), with a total of 1,697 differential ion features were selected (Figure 4C). There were 26 differential metabolites were annotated that could significantly separate the cancerous and normal tissue samples (Figure 4D). However, analysis identified only kynurenine and LPC(14:0)sn-1 of the four prognostic circulating metabolites. Significantly higher levels of kynurenine and LPC(14:0)sn-1 were observed in cancerous tissues compared with normal counterparts (Figures 4E,F). Interestingly, when other molecules involved in tryptophan-kynurenine metabolism were investigated, the levels of tryptophan and formylkynurenine were also found to be higher in cancerous tissues (Figures 4G,H). Although hippuric acid was not detected by tissue metabolomic analysis, phenylalanine, a potential parent metabolite of hippuric acid, was found at higher levels in cancerous tissues (Figure 4I). ROC curve analysis showed that tissue formylkynurenine levels had the best diagnostic performance (max AUC_ROC_: 0.957), followed by kynurenine (AUC_ROC_:0.947), LPC(14:0)sn-1 (AUC_ROC_: 0.875), phenylalanine (AUC_ROC_: 0.856), and tryptophan (AUC_ROC_: 0.762) (Figure 4J). Taken together, tissue-based metabolomics inferred altered kynurenine pathway to be the most significant feature of ESCC.

### IDO1 Up-Regulation in ESCC

**Figure 5:**
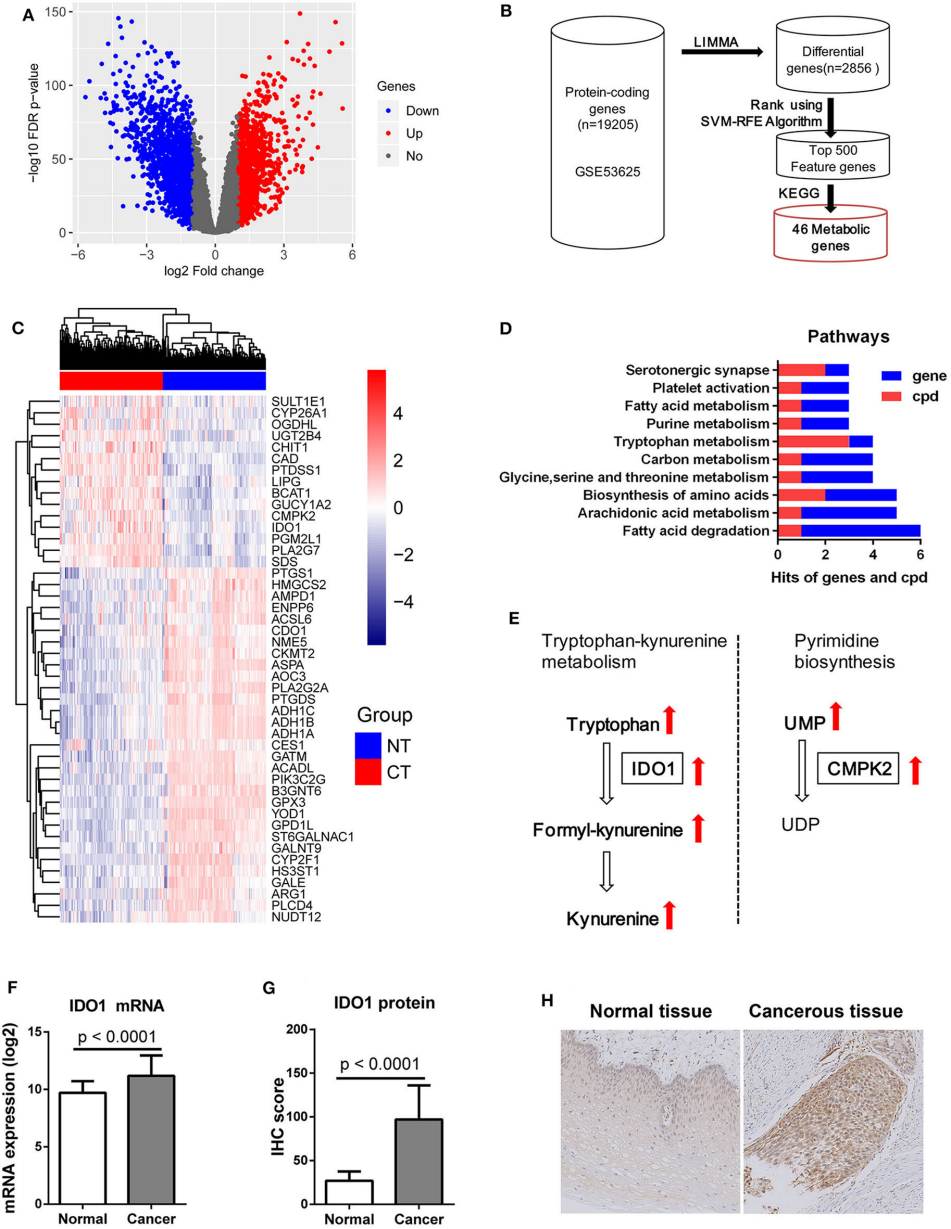
IDO1 expression in ESCC. **(A)** Volcano plot of 2856 DEGs with |log_2_(Fold change)| > 1 and FDR < 0.05 for transcriptomic data from 179 paired ESCC tissue samples from GEO microarray data (GSE53625), red dot: up-regulated gene, blue dot: down-regulated gene. **(B)** Schematic diagram for feature metabolic gene selection process. **(C)** Heatmap with 46 feature metabolic genes, NT: normal tissue, CT: cancerous tissue. **(D)** Top 10 metabolism pathways with both hits for gene and metabolite (cpd). **(E)** Significantly altered metabolic modules with both hits for gene and metabolite, UMP, uridine monophosphate; UDP, uridine diphosphate. **(F)** mRNA expression of IDO1 was significantly increased in ESCC cancerous tissues compared to normal counterparts. **(G)** IHC analysis of IDO1 protein expression showing significant up-regulation in cancerous tissues compared to normal counterparts. **(H)** Representative IHC staining of IDO1 in cancerous and matched equivalents, Magnification: 400 ×.

After LIMMA differential expression analysis, a total of 2,856 DEGs were screened (Figure 5A). After SVM-RFE ranking, the top 500 genes were then selected as feature genes, including 46 metabolic genes (Figure 5B). The 46 metabolic feature genes were able to separate the cancerous and normal tissues (Figure 5C). Pathway analysis of both the 46 DEGs and the above 26 metabolites identified the top 10 metabolic pathways, in which fatty acid degradation ranked first in total hits/gene hits, and tryptophan metabolism had the most metabolite hits (Figure 5D). By module analysis, two potential modules, kynurenine pathway (metabolite: tryptophan, formylkynurenine, kynurenine, and gene: *IDO1*) and pyrimidine biosynthesis (metabolite: uridine monophosphate (UMP) and uridine diphosphate (UDP); gene: *CMPK2*), were identified by criteria (1) with both gene and metabolite hits, and (2) direct interaction between gene and metabolite (Figure 5E). Based on these results: (1) Kynurenine in plasma was associated with OS of patients with ESCC. (2) Significant accumulation of tryptophan, kynurenine, and formlykynurenine was found in ESCC cancerous tissue. (3) Upregulation of *IDO1* mRNA, which was the 209th feature in the 2856 DEGs by SVM-RFE ranking, and the 18th one in the 46 metabolic feature genes, was found in cancerous compared to normal tissues with a high level of statistical significance (Figure 5F). Consistent with these observations, IHC analysis demonstrated significantly higher levels of IDO1 in cancerous tissues compared to paired normal counterparts (Figures 5G,H). Collectively, kynurenine pathway is an important metabolic feature of ESCC, with IDO1 as a potential therapeutic target.

## Discussion

The present study revealed that circulating kynurenine, LPC(14:0)sn-1, 2-piperidinone, and hippuric acid were prognostic factors for ESCC. A kynurenine and LPC(14:0)sn-1 based risk score significantly improved the prediction accuracy of the current TNM staging system in ESCC. Up-regulated tryptophan pathway metabolism, including the accumulation of tryptophan, formylkynurenine, and kynurenine, as well as increased expression of IDO1, were identified as the most significant metabolic features of ESCC.

Circulating-metabolite-based prognostic models have previously been shown to have its promising clinical applications in several cancers, such as glioblastoma (34), non-small cell lung cancer (35), and esophageal adenocarcinoma (24). However, the previous metabolomic studies of ESCC solely focused on the diagnostic value of the metabolites and rarely assessed their prognostic significance. This study is the first to investigate the prognostic value of plasma metabolites in ESCC and found several metabolic biomarkers as well as established a metabolite-based risk score for ESCC. For this study, a combination of circulating metabolomic profiling and survival analysis was used to develop a prognostic approach for ESCC, which identified the kynurenine and LPC(14:0)sn-1 based risk score to have prognostic significance for ESCC. By use of the risk score, ESCC patients were stratified by risk within the same TNM stage (TNM II and III) or the same N stage (N1 and N2). As such, the risk score may assist clinical decision-making, leading to a better prognosis for ESCC patients. It is important to note that due to the limited sample size of TNM subgroups, TNM I (*n* =23) and TNM IV (*n* = 24), it was unavailable to evaluate its risk stratification for these subgroups. Further, for N0 classification, a trend based on risk score was observed that was not statistically significant (*p* = 0.086) (*n* = 101). Future analysis with larger cohorts is essential to determine the clinical significance of the risk score for these subgroups.

Kynurenine was the most interesting circulating biomarker identified by this study. It is one of the main metabolites of tryptophan metabolism, which is related to immune homeostasis, and is correlated with cancer initiation and development (12). Previously, Cheng et al. found the ratio of kynurenine/tryptophan in plasm to be significantly increased in ESCC, and correlated with lymph node metastasis. However, the relationship between kynurenine levels and their survival outcome were not considered (18). To the best of our knowledge, our study is the first to identify circulating kynurenine as a prognostic factor for ESCC, in which higher levels of kynurenine were correlated with poorer OS, and higher N stage and tumor grade levels. Our study and the previous study by Cheng at al. collectively indicate that circulating kynurenine is a promising unfavorable prognostic biomarker for ESCC. The negative correlation of kynurenine levels with survival outcomes is consistent with the immune suppressor role of kynurenine in cancers, in which many cancers enhance kynurenine levels by up-regulating IDOs activity, resulting in escape from immune clearance (36).

Our tissue-based metabolomics revealed up-regulated kynurenine pathway is a significant feature of ESCC, including the accumulation of kynurenine, tryptophan, and formylkynurenine. Significant accumulation of tryptophan and kynurenine in ESCC was reported previously by Tokunaga et al. (23) and Zhang et al. (22), respectively, with this study the first to identify up-regulated formylkynurenine in ESCC. The fold change of formylkynurenine in ESCC was 11.9 and displayed the best diagnostic performance in this study. Formylkynurenine is the direct metabolite of tryptophan mediated by IDO, with increased accumulation of formylkynurenine resulting in the production of the immune suppressive metabolite, kynurenine (fold change of 5.7), in ESCC cancer tissue. Therefore, up-regulated kynurenine pathway not only explained (at least partially) the increased serum kynurenine levels in ESCC patients reported by Cheng et al. (18), but also implied an important role for kynurenine in ESCC progression. In addition to the metabolite level, our bioinformatics analysis and IHC staining analysis demonstrated the key rate-limiting enzyme, IDO1, to be significantly up-regulated in ESCC, which could be a potential therapeutic target for ESCC. Recently, Liu et al. found that kynurenine could up-regulate PD-1 expression on tumor infiltrating T cells through the IDO-kynurenine-AhR pathway (37). The significant accumulation of kynurenine in ESCC suggests that IDO1 inhibitors in combination with other immunotherapies (such as anti-PD-1/anti-PD-L1) may be useful as future therapeutics for ESCC.

LPC(14:0)sn-1, another prognostic circulating metabolite, is a form of lysophosphatidylcholine, in which a phosphorylcholine moiety occupies a glycerol substitution site. Since there are co-existence of LPC(14:0)sn-1 with myristic acid at the C-1 position (sn-1) and LPC(14:0)sn-2 with myristic acid at C-2 position (sn-2) in plasma and these two LPC(14:0)s have the same molecular weight and similar retention time by chromatography, it is crucial to identify LPC(14:0)sn-1 with standard compound. Xu et al. previously reported the down-regulation of plasma LPC(14:0) in ESCC, but they did not clarify the exact position of myristic acid in LPC(14:0) since the study only employed database searches. We believe our study is the first to identify LPC(14:0)sn-1 as a potential prognostic biomarker for ESCC. In the previous study by Xu et al. LPC(14:0) was shown to be a diagnostic plasma metabolite for ESCC. Our study for the first time identified the prognostic potential of circulating LPC(14:0)sn-1 for ESCC in that patients with higher levels had longer OS. Our tissue-based metabolomic analysis detected a significant accumulation of LPC(14:0)sn-1 in cancerous tissue, indicating up-regulated lipid metabolism in ESCC (at least within the lysophosphatidylcholine metabolism pathway). Kamphorst et al. demonstrated cancer cells to directly uptake and use lipids from circulation by macropinocytosis (38, 39). We propose that alterations in circulating LPC(14:0)sn-1 might be associated with enhanced lipid consumption by cancer cells. However, the reason why ESCC patients with lower LPC(14:0)sn-1 have poorer OS is unknown, and further study is needed to explore the potential biological functions of LPC(14:0)sn-1 in ESCC.

2-piperidinone and hippuric acid are the other two prognostic circulating metabolites only detected in plasma in this study. 2-piperidinone was previously found to be decreased in plasma of patients with ovarian cancer (40), but first identified in ESCC. Hippuric acid is formed by the conjugation of benzoic acid with glycine and it is an end-product of phenylalanine metabolism (41). Since increased uptake of phenylalanine was observed in ESCC cancerous tissues, a potential relationship between phenylalanine metabolism and changes in circulating hippuric acid can be postulated. However, neither 2-piperidinone nor hippuric acid were detected by tissue analysis, which suggests that the two metabolites may not have originated from cancer cells. The detailed origin and biological activity of these two metabolites requires further investigation.

Nevertheless, limitations of this study must be considered. First, the biological activity of the four circulating prognostic metabolites, in particular kynurenine and LPC(14:0)sn-1, has not been clarified. Additionally, there are limitations in metabolite annotation and identification, which is a common problem for all metabolomic studies. Future efforts are required to resolve this issue for the entire field.

In conclusion, after identification of potential candidates for circulating prognostic metabolites, and validation by risk score based on plasma levels and correlation coefficients, kynurenine and LPC(14:0)sn-1 were identified as two circulating metabolite biomarkers with prognostic potential. The identified risk score significantly improved prediction accuracy of the TNM staging system and allowed better stratification of ESCC clinical risk. This study demonstrated kynurenine pathway dysregulation in ESCC, which was accompanied by upregulation of IDO1. These observations provide novel insights into the molecular mechanisms of ESCC tumorigenesis and the possible identification of therapeutic targets for ECSS.

## Data Availability Statement

The datasets generated for this study can be found in the GEO/GSE53625/ https://www.ncbi.nlm.nih.gov/geo/query/acc.cgi?acc=GSE53625.

## Ethics Statement

The studies involving human participants were reviewed and approved by the Research Ethics Committee of Zhejiang Cancer Hospital, China. The patients/participants provided their written informed consent to participate in this study. Written informed consent was obtained from the individual(s) for the publication of any potentially identifiable images or data included in this article.

## Author Contributions

ZC, SZ, and WM conceived, designed the study, interpreted the data, wrote the first draft of the manuscript, and contributed to the final version of the manuscript. YD, XH, and YG performed the experiment. KC helped to interpret the data and write the manuscript. NL, DW, and AC helped to collect the clinical data and perform the statistical analysis. QY conducted the bioinformatics analysis. YH instructed the metabolomics analysis. All authors contributed to the article and approved the submitted version.

## Conflict of Interest

The authors declare that the research was conducted in the absence of any commercial or financial relationships that could be construed as a potential conflict of interest.
