# Supplementary material for: Combined Metabolomic Analysis of Plasma and Tissue Reveals a Prognostic Risk Score System and Metabolic Dysregulation in Esophageal Squamous Cell Carcinoma
Source: Front Oncol. 2020 Aug 26;10:1545. doi: 10.3389/fonc.2020.01545 (PMC7479226; doi:10.3389/fonc.2020.01545)
Supplement: Supplementary file 1 [file Table_1.DOCX]

**Table S1:** Gradient elution conditions

| **Time (min)** | **Flow (mL/min)** | **0.1% Formic acid** | **Acetonitrile** |
| --- | --- | --- | --- |
| 0.0 | 0.3 | 98.0 | 2.0 |
| 1.0 | 0.3 | 98.0 | 2.0 |
| 10.0 | 0.3 | 0.0 | 100.0 |
| 13.0 | 0.3 | 0.0 | 100.0 |
| 13.1 | 0.3 | 98.0 | 2.0 |
| 16.0 | 0.3 | 98.0 | 2.0 |

**Table S2:** Mass spectrometer parameters for MS and MS/MS modes

| **Parameters** | **MS** | | **MS/MS** | |
| --- | --- | --- | --- | --- |
|  | **Positive** | **Negative** | **Positive** | **Negative** |
| **Capillary temperature(kv)** | 3.5 | 2.5 | 3.5 | 2.5 |
| **Sheath gas (N2) flow rate** | 35 | 40 | 35 | 40 |
| **Auxiliary gas (N2) flow rate** | 10 | 8 | 10 | 8 |
| **Probe heater temperature(◦C)** | 320 | 350 | 320 | 350 |
| **S-Lens RF level** | 55 | 55 | 55 | 55 |
| **Automatic gain control (AGC)ions** | 1 × 10^6^ | 1 × 10^6^ | 1 × 10^6^ | 1 × 10^6^ |
| **Maximum ion injection time (IT) (ms)** | 100 | 100 | 100 | 100 |
| **Mass data** | Centroid | Centroid | Centroid | Centroid |
| **Mass range** | 70-1000 | 70-1000 | 70-1000 | 70-1000 |
| **Resolution** | 70000 | 70000 | 70000 | 70000 |
| **Collision energies(ev)** | - | - | 10, 20, 40 | 10, 20, 40 |

**Table S3:** Plasma metabolites identified by Compound Discoverer 3.0 in positive mode

| **No.** | **Metabolites** | **Formula** | **W.M. ^a^** | **Ion** | **m/z ^b^** | **RT(min) ^c^** | |
| --- | --- | --- | --- | --- | --- | --- | --- |
| 1 | 2-Piperidinone | C5H9NO | 99.06841 | [M+H]+1 | 100.0763 | 3.9 | |
| 2 | L-(-)-Serine | C3H7NO3 | 105.0427 | [M+H]+1 | 106.0504 | 0.877 | |
| 3 | Uracil | C4H4N2O2 | 112.02782 | [M+H]+1 | 113.0351 | 1.99 | |
| 4 | Creatinine | C4H7N3O | 113.05935 | [M+H]+1 | 114.0666 | 0.932 | |
| 5 | L-Histidine | C6H9N3O2 | 114.04328 | [M+H]+1 | 115.0508 | 1.17 | |
| 6 | Proline | C5H9NO2 | 115.06371 | [M+H]+1 | 116.071 | 0.956 | |
| 7 | Levulinic acid | C5H8O3 | 116.04785 | [M+H]+1 | 117.0551 | 0.891 | |
| 8 | Indole | C8H7N | 117.05811 | [M+H]+1 | 118.0655 | 4.603 | |
| 9 | Betaine | C5H11NO2 | 117.07936 | [M+H]+1 | 118.0866 | 0.893 | |
| 10 | Valine | C5H11NO2 | 117.07937 | [M+H]+1 | 118.0866 | 1.141 | |
| 11 | 2,4,6-Octatriyn-1-ol | C8H6O | 118.04225 | [M+H]+1 | 119.0495 | 2.072 | |
| 12 | L-(-)-Threonine | C4H9NO3 | 119.05861 | [M+H]+1 | 120.0659 | 0.891 | |
| 13 | 4-Vinylphenol | C8H8O | 120.05776 | [M+H]+1 | 121.065 | 8.45 | |
| 14 | Benzoic acid | C7H6O2 | 122.03714 | [M+H]+1 | 123.0444 | 2.07 | |
| 15 | 6-Acetyl-2,3,4,5-tetrahydropyridine | C7H11NO | 125.0844 | [M+H]+1 | 126.0917 | 4.367 | |
| 16 | L-Pyroglutamic acid | C5H7NO3 | 129.04284 | [M+H]+1 | 130.0501 | 1.173 | |
| 17 | Pipecolic acid | C6H11NO2 | 129.07924 | [M+H]+1 | 130.0865 | 1.152 | |
| 18 | Creatine | C4H9N3O2 | 131.06966 | [M+H]+1 | 132.0769 | 0.952 | |
| 19 | Isoleucine | C6H13NO2 | 131.09483 | [M+H]+1 | 132.1021 | 2.051 | |
| 20 | Leucine | C6H13NO2 | 131.09483 | [M+H]+1 | 132.1021 | 2.208 | |
| 21 | 2-Aminoacetophenone | C8H9NO | 135.06862 | [M+H]+1 | 136.0759 | 2.069 | |
| 22 | Hypoxanthine | C5H4N4O | 136.03873 | [M+H]+1 | 137.046 | 1.651 | |
| 23 | Methylimidazoleacetic acid | C6H8N2O2 | 140.05873 | [M+H]+1 | 141.066 | 1.011 | |
| 24 | 6-Methylquinoline | C10H9N | 143.07367 | [M+H]+1 | 144.0809 | 4.604 | |
| 25 | DL-Stachydrine | C7H13NO2 | 143.09482 | [M+H]+1 | 144.1021 | 0.982 | |
| 26 | 4-Indolecarbaldehyde | C9H7NO | 145.05292 | [M+H]+1 | 146.0601 | 4.603 | |
| 27 | Acetylcholine | C7H15NO2 | 145.11036 | [M+H]+1 | 146.1176 | 0.958 | |
| 28 | Coumarin | C9H6O2 | 146.03693 | [M+H]+1 | 147.0442 | 2.048 | |
| 29 | L-Glutamine | C5H10N2O3 | 146.06929 | [M+H]+1 | 147.0765 | 0.87 | |
| 30 | DL-Lysine | C6H14N2O2 | 146.10565 | [M+H]+1 | 147.1129 | 0.769 | |
| 31 | Methionine | C5H11NO2S | 149.05124 | [M+H]+1 | 150.0585 | 1.461 | |
| 32 | DL-α-Aminocaprylic acid | C8H17NO2 | 159.12615 | [M+H]+1 | 160.1333 | 5.185 | |
| 33 | DL-Carnitine | C7H15NO3 | 161.10528 | [M+H]+1 | 162.1125 | 0.91 | |
| 34 | 2-Hydroxycinnamic acid | C9H8O3 | 164.0476 | [M+H]+1 | 165.0549 | 2.081 | |
| 35 | 3-phenyllactic acid | C9H10O3 | 166.06325 | [M+H]+1 | 167.0705 | 5.733 | |
| 36 | Uric acid | C5H4N4O3 | 168.02852 | [M+H]+1 | 169.0358 | 1.55 | |
| 37 | 1-Methylhistidine | C7H11N3O2 | 169.08528 | [M+H]+1 | 170.0926 | 0.864 | |
| 38 | N-2-Acetyl-L-ornithine | C7H14N2O3 | 174.10069 | [M+H]+1 | 175.108 | 1.007 | |
| 39 | DL-Arginine | C6H14N4O2 | 174.11187 | [M+H]+1 | 175.1191 | 0.841 | |
| 40 | N-Amidino-L-aspartic acid | C5H9N3O4 | 175.0587 | [M+H]+1 | 176.066 | 2.144 | |
| 41 | Indole-3-acetic acid | C10H9NO2 | 175.06355 | [M+H]+1 | 176.0709 | 6.508 | |
| 42 | DL-Citrulline | C6H13N3O3 | 175.09583 | [M+H]+1 | 176.1031 | 0.907 | |
| 43 | 5-methylthioribose | C6H12O4S | 180.04577 | [M+H]+1 | 181.0531 | 1.135 | |
| 44 | L-Tyrosine | C9H11NO3 | 181.0741 | [M+H]+1 | 182.0814 | 2.05 | |
| 45 | 3,4-Dihydroxy-5-methoxybenzoic acid | C8H8O5 | 184.03743 | [M+H]+1 | 185.0447 | 5.751 | |
| 46 | Aceglutamide | C7H12N2O4 | 188.07987 | [M+H]+1 | 189.0872 | 0.981 | |
| 47 | 5-Hydroxyindole-3-acetic acid | C10H9NO3 | 191.05844 | [M+H]+1 | 192.0657 | 3.871 | |
| 48 | 2,6-Diisopropyl-3-methylphenol | C13H20O | 192.15167 | [M+H]+1 | 193.159 | 8.026 | |
| 49 | Acetyl-L-carnitine | C9H17NO4 | 203.11598 | [M+H]+1 | 204.1233 | 1.43 | |
| 50 | trans-3-Indoleacrylic acid | C11H9NO2 | 204.09001 | [M+H]+1 | 205.0972 | 4.604 | |
| 51 | Indole-3-lactic acid | C11H11NO3 | 205.07416 | [M+H]+1 | 206.0815 | 6.027 | |
| 52 | Kynurenine | C10H12N2O3 | 208.08479 | [M+H]+1 | 209.0922 | 3.9 | |
| 53 | Propionylcarnitine | C10H19NO4 | 217.13164 | [M+H]+1 | 218.1388 | 2.789 | |
| 54 | Nootkatone | C15H22O | 218.16721 | [M+H]+1 | 219.1745 | 7.864 | |
| 55 | D-PANTOTHENIC ACID | C9H17NO5 | 219.11075 | [M+H]+1 | 220.118 | 4.218 | |
| 56 | dillapiole | C12H14O4 | 222.08931 | [M+H]+1 | 223.0966 | 7.613 | |
| 57 | N,N'-Dicyclohexylurea | C13H24N2O | 224.18898 | [M+H]+1 | 225.1963 | 8.288 | |
| 58 | L-Ergothioneine | C9H15N3O2S | 229.08874 | [M+H]+1 | 230.0961 | 1.003 | |
| 59 | C4-Carnitine | C11H21NO4 | 231.14727 | [M+H]+1 | 232.1547 | 4.397 | |
| 60 | thr-asp | C8H14N2O6 | 234.08532 | [M+H]+1 | 235.0926 | 0.981 | |
| 61 | Formylkynurenine | C11H12N2O4 | 236.07988 | [M+H]+1 | 237.087 | 4.053 | |
| 62 | 3,4,5-trimethoxyhydrocinnamic acid | C12H16O5 | 240.09995 | [M+H]+1 | 241.1071 | 7.613 | |
| 63 | N-Undecanoylglycine | C13H25NO3 | 243.18357 | [M+H]+1 | 244.1909 | 7.316 | |
| 64 | 4-Hydroxyprolylleucine | C11H20N2O4 | 244.14253 | [M+H]+1 | 245.1498 | 5.462 | |
| 65 | 2-Hexenoylcarnitine | C13H23NO4 | 257.16304 | [M+H]+1 | 258.1703 | 5.397 | |
| 66 | Hexanoylcarnitine | C13H25NO4 | 259.17859 | [M+H]+1 | 260.1859 | 5.685 | |
| 67 | gamma-Glutamylleucine | C11H20N2O5 | 260.1375 | [M+H]+1 | 261.1448 | 4.664 | |
| 68 | Capsi-amide | C17H35NO | 269.27214 | [M+H]+1 | 270.2794 | 13.873 | |
| 69 | Acetylpterosin C | C16H20O4 | 276.13646 | [M+H]+1 | 277.1438 | 8.844 | |
| 70 | Amide C18 | C18H37NO | 283.28769 | [M+H]+1 | 284.2949 | 14.368 | |
| 71 | Methyl palmitate | C17H34O2 | 287.28258 | [M+H]+1 | 288.2899 | 7.936 | |
| 72 | 1-Hexadecanoylpyrrolidine | C20H39NO | 309.30329 | [M+H]+1 | 310.3106 | | 13.079 |
| 73 | 9-Decenoylcarnitine | C17H31NO4 | 313.22548 | [M+H]+1 | 314.2325 | 7.216 | |
| 74 | Decanoylcarnitine | C17H33NO4 | 315.24106 | [M+H]+1 | 316.2483 | 7.396 | |
| 75 | trans-2-Dodecenoylcarnitine | C19H35NO4 | 341.25684 | [M+H]+1 | 342.2642 | 7.731 | |
| 76 | Trp-Phe | C20H21N3O3 | 351.15846 | [M+H]+1 | 352.1658 | 5.724 | |
| 77 | Cortisol | C21H30O5 | 362.20954 | [M+H]+1 | 363.2169 | 6.868 | |
| 78 | 3,5-Tetradecadiencarnitine | C21H37NO4 | 367.27253 | [M+H]+1 | 368.2799 | 8.036 | |
| 79 | Palmitoylcarnitine | C23H45NO4 | 399.33508 | [M+H]+1 | 400.3423 | 9.308 | |
| 80 | Linoleylcarnitine | C25H45NO4 | 423.33503 | [M+H]+1 | 424.3422 | 9.086 | |
| 81 | O-oleoylcarnitine | C25H47NO4 | 425.35066 | [M+H]+1 | 426.3579 | 9.448 | |
| 82 | 4alpha-formyl-4beta-methyl-5alpha-8-cholesten-3beta-ol | C29H48O2 | 428.36591 | [M+H]+1 | 429.3733 | 14.27 | |
| 83 | Hecogenin | C27H42O4 | 430.30887 | [M+H]+1 | 431.3162 | 10.166 | |
| 84 | Glycochenodeoxycholic acid | C26H43NO5 | 449.31451 | [M+H]+1 | 450.3217 | 8.301 | |
| 85 | LPC(14:0) | C22H46NO7P | 467.30139 | [M+H]+1 | 468.3088 | 8.923 | |
| 86 | LPC(16:2e) | C24H48NO6P | 477.3212 | [M+H]+1 | 478.3278 | 15.13 | |
| 87 | LPE(18:0/0:0) | C23H48NO7P | 481.31713 | [M+H]+1 | 482.3243 | 10.885 | |
| 88 | LPC(16:0e) | C24H52NO6P | 481.3525 | [M+H]+1 | 482.3592 | 10.13 | |
| 89 | LPC(16:1) | C24H48NO7P | 493.3161 | [M+H]+1 | 494.3227 | 9.19 | |
| 90 | LPC(16:0) | C24H50NO7P | 495.3318 | [M+H]+1 | 496.3381 | 9.88 | |
| 91 | LPC(17:1) | C25H50NO7P | 507.3318 | [M+H]+1 | 508.3387 | 9.67 | |
| 92 | LPC(P-18:0) | C26H54NO6P | 507.36935 | [M+H]+1 | 508.3767 | 10.425 | |
| 93 | LPC(17:0) | C25H52NO7P | 509.3474 | [M+H]+1 | 510.354 | 10.37 | |
| 94 | LPC(18:3) | C26H48NO7P | 517.3161 | [M+H]+1 | 518.3225 | 9.14 | |
| 95 | LPC(18:2) | C26H50NO7P | 519.33295 | [M+H]+1 | 520.3402 | 9.527 | |
| 96 | LPC(18:1) | C26H52NO7P | 521.3474 | [M+H]+1 | 522.3539 | 10.17 | |
| 97 | LPC(18:0) | C26H54NO7P | 523.36409 | [M+H]+1 | 524.3714 | 10.961 | |
| 98 | LPC(20:5) | C28H48NO7P | 541.31723 | [M+H]+1 | 542.3245 | 9.072 | |
| 99 | LPC(20:4) | C28H50NO7P | 543.3318 | [M+H]+1 | 544.3383 | 9.4 | |
| 100 | LPC(20:3) | C28H52NO7P | 545.3474 | [M+H]+1 | 546.3537 | 9.86 | |
| 101 | LPC(20:2) | C28H54NO7P | 547.3631 | [M+H]+1 | 548.3691 | 10.39 | |
| 102 | LPC(20:1) | C28H56NO7P | 549.3787 | [M+H]+1 | 550.3852 | 11.16 | |
| 103 | LPC(22:6) | C30H50NO7P | 567.3318 | [M+H]+1 | 568.3379 | 9.53 | |
| 104 | LPC(22:5) | C30H52NO7P | 569.34867 | [M+H]+1 | 570.356 | 9.745 | |
| 105 | LPC(22:4) | C30H54NO7P | 571.3631 | [M+H]+1 | 572.3694 | 10.13 | |

^a^: molecular weight; ^b^: m/z: mass/charge number; ^c^: retention time.

**Table S4:** Plasma metabolites identified by Compound Discoverer 3.0 in negative mode

| **No.** | **Metabolites** | **Formula** | **M.W. ^a^** | **Ion** | **m/z ^b^** | **RT(min) ^c^** | |
| --- | --- | --- | --- | --- | --- | --- | --- |
| 1 | Pyruvic acid | C3H4O3 | 88.0152 | [M-H]-1 | 87.0072 | 1.14 |  |
| 2 | DL-Lactic Acid | C3H6O3 | 90.03084 | [M-H]-1 | 89.02284 | 1.2 |  |
| 3 | 3-Hydroxybutyric acid | C4H8O3 | 104.04664 | [M-H]-1 | 103.0386 | 2.63 |  |
| 4 | 5-Aminovaleric acid | C5H11NO2 | 117.07844 | [M-H]-1 | 116.0704 | 1.191 |  |
| 5 | 2-Hydroxyisovaleric acid | C5H10O3 | 118.06241 | [M-H]-1 | 117.0544 | 4.552 |  |
| 6 | 6-Oxohexanoic acid | C6H10O3 | 130.06256 | [M-H]-1 | 129.0546 | 5.342 |  |
| 7 | L-Threonic acid | C4H8O5 | 136.03682 | [M-H]-1 | 135.0288 | 0.96 |  |
| 8 | Hypoxanthin | C5H4N4O | 136.0383 | [M-H]-1 | 135.0303 | 1.2 |  |
| 9 | D-(-)-Glutamine | C5H10N2O3 | 146.06894 | [M-H]-1 | 145.0609 | 0.913 |  |
| 10 | Suberic acid | C8H14O4 | 174.08954 | [M-H]-1 | 173.0815 | 5.716 |  |
| 11 | Hippuric acid | C9H9NO3 | 179.05865 | [M-H]-1 | 178.0507 | 5.241 |  |
| 12 | DL-4 Hydroxyphenyllactic  acid | C9H10O4 | 182.05836 | [M-H]-1 | 181.0504 | 4.696 |  |
| 13 | p-Cresylsulfate | C7H8O4S | 188.01488 | [M-H]-1 | 187.0069 | 6.008 |  |
| 14 | 3-Hydroxydecanoic acid | C10H20O3 | 188.1418 | [M-H]-1 | 187.1338 | 8.028 |  |
| 15 | DL-Tryptophan | C11H12N2O2 | 204.09076 | [M-H]-1 | 203.0828 | 4.612 |  |
| 16 | 1,3,7-Trimethyluric acid | C8H10N4O3 | 210.07487 | [M-H]-1 | 209.0669 | 1.013 |  |
| 17 | 3-Indoxyl sulphate | C8H7NO4S | 213.01062 | [M-H]-1 | 212.0026 | 5.481 |  |
| 18 | Casimiroin | C12H11NO4 | 233.06785 | [M-H]-1 | 232.0599 | 3.982 |  |
| 19 | CMPF | C12H16O5 | 240.1013 | [M-H]-1 | 239.0933 | 7.629 |  |
| 20 | Uridine | C9H12N2O6 | 244.0712 | [M-H]-1 | 243.0632 | 2.133 |  |
| 21 | Palmitoleic acid | C16H30O2 | 254.22647 | [M-H]-1 | 253.2185 | 11.897 |  |
| 22 | α-Linolenic acid | C18H30O2 | 278.22682 | [M-H]-1 | 277.2188 | 11.559 |  |
| 23 | Oleic acid | C18H34O2 | 282.25815 | [M-H]-1 | 281.2502 | 12.843 |  |
| 24 | Eicosapentaenoic acid | C20H30O2 | 302.22703 | [M-H]-1 | 301.219 | 11.455 |  |
| 25 | Arachidonic acid | C20H32O2 | 304.24263 | [M-H]-1 | 303.2346 | 11.961 |  |
| 26 | 8Z,11Z,14Z-Eicosatrienoic acid | C20H34O2 | 306.25847 | [M-H]-1 | 305.2505 | 12.367 |  |
| 27 | Docosahexaenoic acid | C22H32O2 | 328.2429 | [M-H]-1 | 327.2349 | 11.782 |  |
| 28 | Docosapentaenoic acid | C22H34O2 | 330.25874 | [M-H]-1 | 329.2507 | 12.071 |  |
| 29 | Adrenic acid | C22H36O2 | 332.27434 | [M-H]-1 | 331.2663 | 12.629 |  |
| 30 | Testosterone sulfate | C19H28O5S | 368.16881 | [M-H]-1 | 367.1608 | 8.239 |  |
| 31 | Glycoursodeoxycholic acid | C26H43NO5 | 449.31798 | [M-H]-1 | 448.31 | 8.326 |  |

^a^: molecular weight; ^b^: m/z: mass/charge number; ^c^: retention time.

**Table S5**: Correlation among the four metabolites

| **Metabolites** |  | **Kynurenine** | **LPC(14:0)sn-1** | **2-Piperidinone** | **Hippuric acid** |
| --- | --- | --- | --- | --- | --- |
| **Kynurenine** | r ^a^ | 1.000 | **0.175**** | **0.181**** | **0.158**** |
|  | p ^b^ | . | 0.004 | 0.003 | 0.009 |
| **LPC(14:0)sn-1** | r | **0.175**** | 1.000 | 0.038 | -0.067 |
|  | p | 0.004 | . | 0.529 | 0.270 |
| **2-Piperidinone** | r | **0.181**** | 0.038 | 1.000 | **0.204**** |
|  | p | 0.003 | 0.529 | . | 0.001 |
| **Hippuric acid** | r | **0.158**** | -0.067 | **0.204**** | 1.000 |
|  | p | 0.009 | 0.270 | 0.001 | . |

^a^: correlation coefficient, ^b^: p value, *****: p ≤ 0.05, ******: p ≤ 0.01.

**Table S6**: Correlation analysis of circulating metabolites with clinicopathologic features and biochemical parameters in patients with ESCC

| **Parameters ^a^** |  | **Metabolites** | | | |
| --- | --- | --- | --- | --- | --- |
|  |  | **Kynurenine** | **LPC(14:0)sn-1** | **2-Piperidinone** | **Hippuric acid** |
| **GPDA** | r ^b^ | -0.083 | **0.145*** | -0.03 | **0.135*** |
|  | p ^c^ | 0.169 | 0.017 | 0.616 | 0.026 |
| **GGT** | r | **0.129*** | 0.109 | **0.142*** | **-0.12*** |
|  | p | 0.033 | 0.072 | 0.019 | 0.048 |
| **ALT** | r | 0.027 | **0.228**** | -0.08 | **-0.123*** |
|  | p | 0.658 | 0 | 0.187 | 0.042 |
| **PA** | r | -0.085 | **0.258**** | 0.008 | **-0.198**** |
|  | p | 0.162 | 0 | 0.891 | 0.001 |
| **ALB** | r | **-0.133*** | 0.114 | 0.012 | -0.067 |
|  | p | 0.027 | 0.058 | 0.843 | 0.269 |
| **TG** | r | 0.043 | **0.175**** | -0.05 | -0.065 |
|  | p | 0.482 | 0.004 | 0.413 | 0.286 |
| **TC** | r | -0.013 | 0.066 | **-0.132*** | -0.043 |
|  | p | 0.831 | 0.272 | 0.028 | 0.474 |
| **HDL-C** | r | -0.042 | **0.155*** | -0.061 | -0.09 |
|  | p | 0.484 | 0.01 | 0.316 | 0.137 |
| **Age** | r | 0.091 | **-0.105*** | -0.067 | 0.041 |
|  | p | 0.065 | 0.033 | 0.174 | 0.402 |
| **BMI** | r | -0.045 | 0.068 | **0.082*** | -0.027 |
|  | p | 0.266 | 0.093 | 0.042 | 0.507 |
| **Smoking habit** | r | 0.076 | 0.044 | 0.040 | -0.065 |
|  | p | 0.124 | 0.373 | 0.422 | 0.187 |
| **Alcohol consumption** | r | 0.054 | 0.049 | **0.116*** | 0.007 |
|  | p | 0.272 | 0.319 | 0.018 | 0.884 |
| **Tumor differentiation** | r | **-0.127**** | 0.004 | -0.007 | -0.035 |
|  | p | 0.008 | 0.941 | 0.884 | 0.471 |
| **N stage** | r | **0.102*** | 0.001 | 0.055 | 0.077 |
|  | p | 0.025 | 0.978 | 0.233 | 0.093 |

^a^: GPDA, glycyl proline dipeptidyl aminopeptidase; GGT, gamma-glutamyltransferase; ALT, alanine aminotransferase; PA, prealbumin; ALB, albumin; TG, triglyceride; TC, total cholesterol; HDL-C, high density lipoprotein cholesterol; BMI, body mass index; ^b^: correlation coefficient; ^c^: p value, *****: p ≤ 0.05, ******: p ≤ 0.05.

**Table S7:** Univariate and multivariate Cox regression analysis for overall survival

| **Factors** | **Univariate Cox** | |  | **Multivariate Cox** | |
| --- | --- | --- | --- | --- | --- |
|  | **HR ^a^** | **p value** |  | **HR** | **p value** |
| **Risk score** | 2.72 | 6.8E-05 |  | 2.32 | 0.001 |
| **Age** | 1.06 | 7.1E-01 |  | - | - |
| **Sex** | 1.02 | 9.4E-01 |  | - | - |
| **Smoking habit** | 1.05 | 7.6E-01 |  | - | - |
| **Alcohol consumption** | 1.27 | 1.6E-01 |  | - | - |
| **Tumor grade** | 0.66 | 3.4E-03 |  | 0.71 | 0.031 |
| **Tumor thrombus** | 1.78 | 3.0E-04 |  | 1.35 | 0.078 |
| **Neural invasion** | 2.01 | 6.4E-06 |  | 1.60 | 0.004 |
| **N stage** | 1.71 | 8.0E-12 |  | 1.64 | 0.002 |
| **T stage** | 1.64 | 3.4E-03 |  | 1.50 | 0.025 |
| **TNM stage** | 2.00 | 4.4E-09 |  | 0.94 | 0.772 |

^a^: hazard ratio.
