# Supplementary figures and images for: Combined Metabolomic Analysis of Plasma and Tissue Reveals a Prognostic Risk Score System and Metabolic Dysregulation in Esophageal Squamous Cell Carcinoma
Source: Front Oncol. 2020 Aug 26;10:1545. doi: 10.3389/fonc.2020.01545 (PMC7479226; doi:10.3389/fonc.2020.01545)

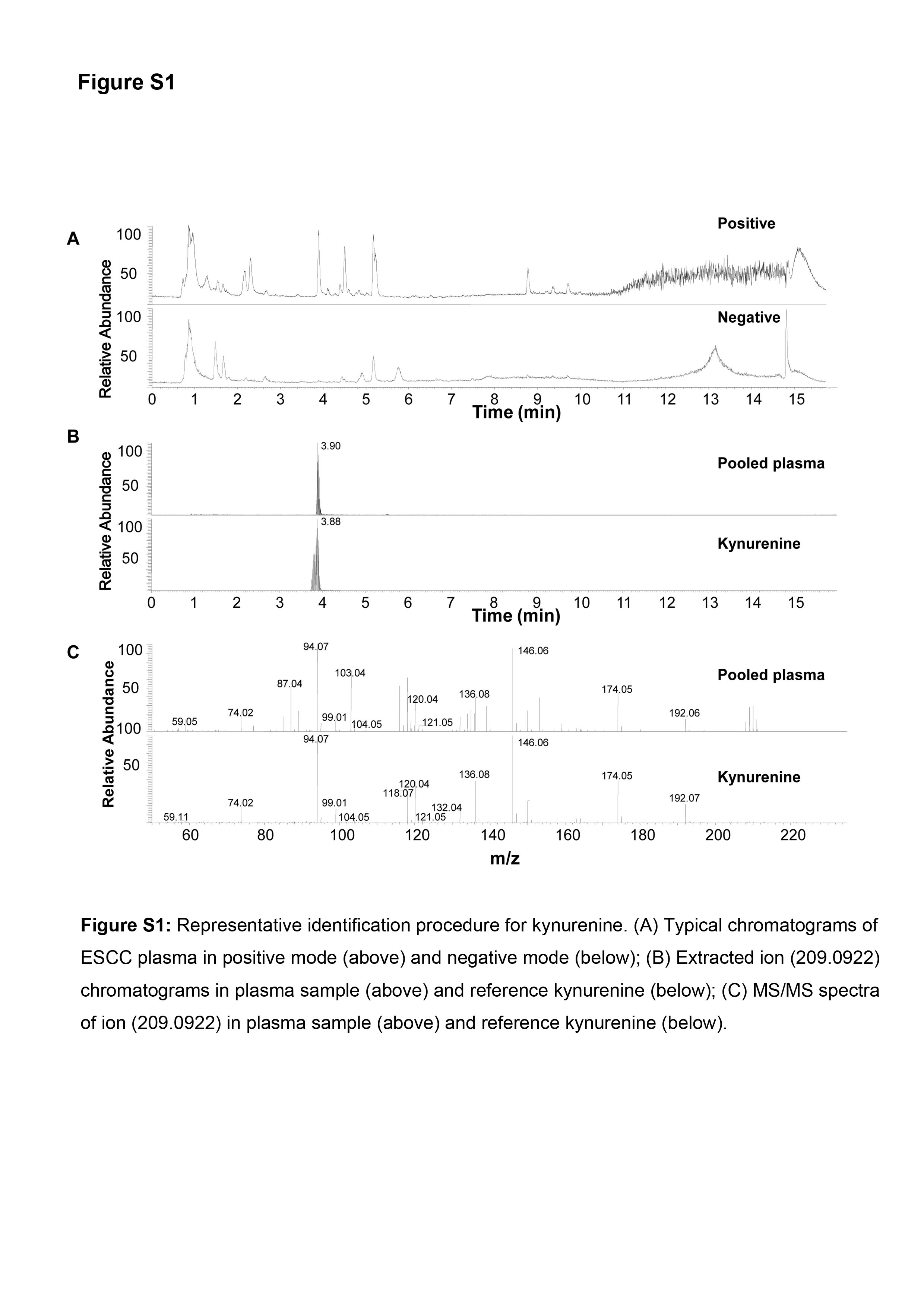

Supplement: Supplementary file 2 [file Image_1.JPEG]

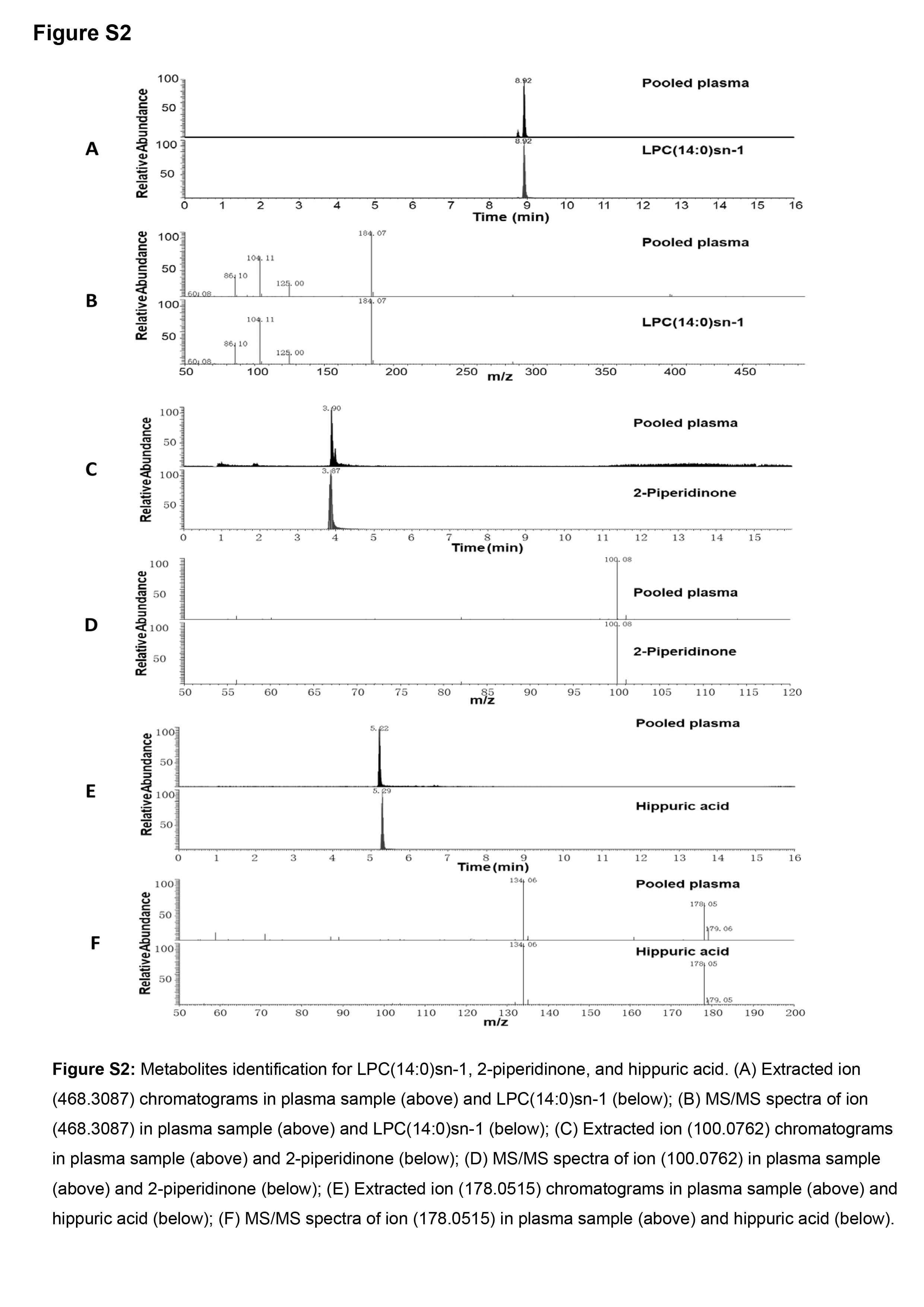

Supplement: Supplementary file 3 [file Image_2.JPEG]

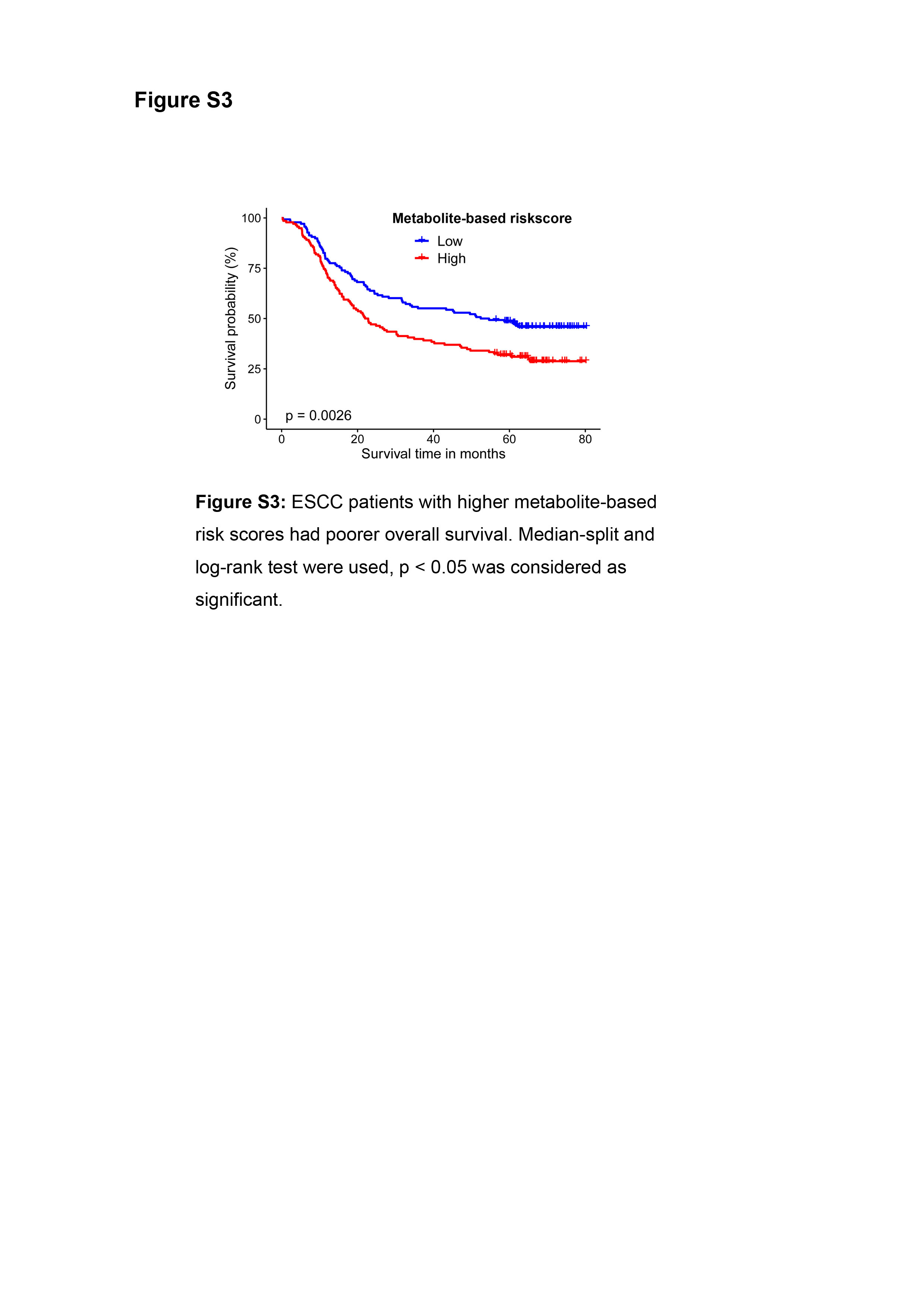

Supplement: Supplementary file 4 [file Image_3.JPEG]
